# The danger of denialism: lessons from the Brazilian pandemic

**DOI:** 10.1186/s42269-021-00516-y

**Published:** 2021-03-09

**Authors:** Heslley Machado Silva

**Affiliations:** University Center of Formiga, Dr. Arnaldo de Senna Avenue, 95, Formiga City, Minas Gerais State 35574-110 Brazil

**Keywords:** Science, Denial, Risks, Vaccine, Pandemic, Brazil, Social media, Conspiracy theories, Public health, Government, Unproven medicines

## Abstract

**Background:**

Scientific denialism has always had harmful consequences for humanity, but with the advent of the pandemic these effects seem to have been accentuated.

**Main body:**

Unwillingness to accept the facts about the COVID-19 pandemic ascertained by scientists and public health authorities has led to widespread scientific denialism, including the emergence of conspiracy theories of all sorts. Examples are diverse, reaching both developed and developing countries, arriving through populist leaders and the spread of conspiracy theories through social media.

**Short conclusion:**

It is important to pay attention to the risk of the extremes of this denialism and the possible repercussions, especially in countries that have leaders who agree with these conceptions, such as Brazil and the USA.

## Background

It is difficult to understand how a relatively educated population like the German people followed the hateful and perverse social ideas of the Nazi government, or how the Soviet people supported Stalin’s anti-science delusion of Lysenkoism, or how the American population agreed to eugenic sterilization (Allen et al. 2001). All these should have remained in the dark past, and we should have learned from these mistakes that generated only suffering—but the COVID-19 pandemic shows us that these demons are not exorcised.

## Main text

It seems unbelievable, but today’s Brazil gives an example of how far radicalism and ignorance can go and how threatening are those who preach and follow scientific denialism, which has found an ideal means of propagation, through social media (McKee and Diethelm 2010). The arrival of a vaccine should have been widely celebrated, especially in a country that has the second largest number of cases of COVID-19. But instead, the first shipment of vaccines to Brazil had to be hidden, not to be within reach of the threat of Brazilian groups that are against vaccination, partly because of fears of its foreign (Chinese) source, and favor supposed alternative remedies of unproven efficacy.

How could this happen? The Brazilian government is complicit. Brazil’s president Jair Bolsonaro recently questioned the need for a rush for a vaccine against COVID-19 (Bello 2020). Lost in daydream, he also stated categorically that he will not take any vaccine, for he mistakenly claims not to need it on the grounds that he would already have been infected by now. Such an attitude could be just a subject for jokes: The government is notorious for its ignorance about many subjects. But its horde of followers is large and noisy, impeding the dissemination of a vaccine that can avert thousands of deaths. And the government’s dismissive attitude toward the COVID-19 vaccine bolsters anti-vaccine movements in general, at a time when vaccination rates for various diseases (such as measles and polio) are decreasing and vaccine rejection rates are increasing around the world (Silva 2020).

Brazil is conspicuous for the virulence of its government-led COVID-19 denialism. But anyone who supposes that this phenomenon is restricted to developing countries would do well to reflect on the death threats received by the American immunologist Anthony Fauci over his role in combating the pandemic there (Ioannidis 2020).

## Conclusions

Scientific denialism is serious and pervasive, and it shows no signs of abatement. Information through the press and the field of education seem to have no effect, becoming instantly discredited by propaganda and populist leaders. The world needs to react to this kind of disturbance, seeking effective ways of confrontation—and the academic field needs to be more active in this difficult battle.

## Data Availability

No.
